# Quantifying the costs of pre‐ and postcopulatory traits for males: Evidence that costs of ejaculation are minor relative to mating effort

**DOI:** 10.1002/evl3.228

**Published:** 2021-05-27

**Authors:** Meng‐Han Joseph Chung, Michael D. Jennions, Rebecca J. Fox

**Affiliations:** ^1^ Division of Ecology and Evolution Research School of Biology Australian National University Canberra ACT 2601 Australia

**Keywords:** Coercive mating, ejaculation, mating effort, Poeciliidae, reproductive costs

## Abstract

Although it is widely stated that both mating behavior and sperm traits are energetically costly for males, we currently lack empirical estimates of the *relative* costs to males of pre‐ versus postcopulatory investments. Such estimates require the experimental separation of the act of mating from that of ejaculation, which is a nontrivial logistical challenge. Here, we overcome this challenge using a novel morphological manipulation (gonopodium tip ablation) in the eastern mosquitofish (*Gambusia holbrooki*) to tease apart investment in mating effort from that in sperm replenishment following ejaculation. We quantified the relative cumulative costs of investing in mating effort and ejaculation by comparing somatic traits and reproductive performance among three types of males: ablated males that could attempt to mate but not ejaculate; unablated males that could both mate *and* ejaculate; and control males that had no access to females. We show that, after eight weeks, mating investment significantly reduces both body growth and immunocompetence and results in a significant decline in mating effort. In contrast, cumulative investment into sperm replenishment following ejaculation has few detectable effects that are only apparent in smaller males. These minor costs occur despite the fact that *G. holbrooki* has very high levels of sperm competition and multiple mating by both sexes, which is usually associated with elevated levels of sperm production. Crucially, our study is the first, to our knowledge, to experimentally compare the relative costs of pre‐ and postcopulatory investment on components of male fitness in a vertebrate.

**Impact Summary**

Investment by males into reproduction (e.g., courtship, nuptial gifts, sperm production) is energy consuming. Costs then arise from a combination of direct mortality, reduced body condition, and/or reproductive senescence. However, it is largely unknown which aspects of male reproduction most strongly contribute to a decline in later‐life performance: males that invest more into mating behavior also invest more into producing ejaculates. Male investments into pre‐ and postcopulatory traits are therefore highly correlated. Here, we experimentally tease apart the costs of male mating activity and ejaculation (and subsequent sperm replenishment) in the mosquitofish (*Gambusia holbrooki*) by surgically removing the tip of the male intromittent organ to prevent ejaculation. This procedure does not itself directly affect male mating behavior. Male *G. holbrooki* only mate following coercive mating attempts and invest heavily into ejaculate traits because females mate with many males. This suggests that the costs of pre‐ and postcopulatory traits can be readily evaluated. We quantified the cost of mating effort by comparing males with and without access to mates, and that of ejaculation by comparing males that could or could not release sperm when interacting with females. These conditions were maintained for eight weeks, equal to half of their breeding season. We show that a decline with age in life history traits (i.e., growth, immune response) is attributed to cumulative investment into mating activity. In contrast, comparable effects of ejaculation (hence sperm replenishment) are negligible: a weaker male preference for larger females and slower sperm replenishment that is only observed in smaller males. Our findings suggest that the cumulative costs of ejaculation are minor compared to those of mating effort, even when sperm competition is intense.

Male reproduction is costly, requiring investment in sexual ornaments and weapons (Emlen 2001), mating behavior (e.g., courtship, mate guarding; Cole and Endler 2016; Dowling and Webster 2017), and sperm/ejaculate production (Olsson et al. 1997). Given these costs, we should expect to see a trade‐off between investing in these sexually selected traits and other activities (e.g. foraging; Garcia and Lemus 2012) that affect key life history parameters under natural selection (e.g., growth, immune function; Gélin et al. 2016; Kulaszewicz et al. 2017; Brokordt et al. 2019). In both sexes the accumulated costs of reproduction tend to lower performance later in life, increase the rate of senescence (Nussey et al. 2006; Reed et al. 2008; Froy et al. 2013), and reduce the lifespan of individuals that mate or breed more often (Miller and Brook 2005; South et al. 2009; Brooks and Garratt 2017). Despite the key role of “costs of reproduction” in life history theory (Williams 1966; Kirkwood and Rose 1991), most studies focus on the fitness costs for females rather than for males (review: Lemaître et al. 2015; Lemaître and Gaillard 2017). Of those studies that do adopt the male perspective, few compare the relative costs of different components of male reproductive effort. For example, it is widely stated that male mating behavior and sperm production are “costly” (e.g., Lüpold et al. 2014; Barbosa et al. 2018; Somjee et al. 2018; Tuni et al. 2018), but is one more costly than the other? And do these costs manifest in different ways, and at different times?

Studies to date have quantified the combined short‐term costs of pre‐ and postcopulatory investments by males (e.g., Griffiths 1996; Gilbert and Uetz 2016), but few have been able to address the issue of the short‐term costs of *individual components* of male reproductive effort, and almost none have quantified the long‐term costs (for exceptions, see Van Voorhies 1992; Cordts and Partridge 1996; Olsson et al. 1997). We therefore currently lack empirical estimates of the *relative* costs for males of investment in precopulatory versus postcopulatory traits, making it very difficult to answer the questions posed above. Part of the reason for the lack of empirical data are the logistical challenges posed by having to tease apart investment in pre‐ and postcopulatory components of reproduction experimentally. In general, a male releases sperm when he acquires a mate and copulates. Investments into mating behaviors and sperm replenishment (following ejaculation) with every successful copulation are therefore confounded. To identify the costs of specific components of male reproductive effort requires an experimental approach in which investment into each component can be independently manipulated. Previous studies that have attempted to tease apart the costs of sperm traits and mating activity have relied either on natural variation among individuals or on patterns of investment over time. For example, the genetic mutation that lowers sperm production in the nematode, *Caenorhabditis elegans* (Van Voorhies 1992), makes it possible to test for potential costs of sperm production by comparing mutant and wildtype males. However, this assumes that the only pleiotropic effects of the mutation on other life history traits are attributable to the change in sperm production. In the European adder, *Vipera berus*, males complete spermatogenesis before commencing mating activity (Olsson et al. 1997), meaning that one can separate the cost of sperm production to quantify the additional cost of mating behavior. Such difference in the timing of pre‐ and postcopulatory investments has also been reported in some ungulates (e.g., Himalayan tahr, feral goats; Ahmad and Noakes 1996; Forsyth et al. 2005) where subadult males pay the cost of producing ejaculates but do not yet engage in mating behavior.

In the eastern mosquitofish (*Gambusia holbrooki*), males attempt to mate with females incessantly (up to an attempt every minute; Wilson 2005), making them ideal for studies of male investment into reproduction. In addition, males mate almost exclusively through coercive copulation attempts and do not provide nuptial gifts or paternal care (Pilastro et al. 1997), meaning that precopulatory mating effort can be readily measured. Females mate multiply (Booksmythe et al. 2016; Head et al. 2017; Gao et al. 2019), with two to three sires per brood in the wild (Zane et al. 1999), so investment in ejaculates is expected to be high due to intense sperm competition (Evans et al. 2003; Lüpold et al. 2020). In this study, we use a validated ablation technique that allows males to attempt to copulate, but renders them unable to ejaculate (Chung et al. 2019, 2020). To achieve this, we remove the tip of the male intromittent organ (a modified anal fin called a “gonopodium”). This minor surgical manipulation accomplishes the necessary separation of mating activity and sperm usage for this species. We have verified that ablated males (1) behave and forage normally within minutes of recovery from surgery (Fox et al. 2019), (2) have the same rate of sperm production as that of intact males (Chung et al. 2019), (3) exhibit normal mating behaviors (i.e., mating attempts, motility, time spent associating with female; Chung et al. 2020), and (4) are equally attractive to females (this study), implying that the inability to transfer sperm after ablation does not affect male motivation to mate. This is also consistent with the assumption made in a recent study using vasectomized versus sham‐vasectomized males to test female precopulatory investment in mice (Garratt et al. 2020). Through the minor morphological manipulation of the male intromittent organ in *G. holbrooki*, we are therefore able to generate males that incur all the costs arising from mating effort (i.e., pursuing females and attempting to forcibly copulate), without incurring the costs of ejaculation (and subsequent sperm replenishment).

To tease apart the costs of mating behavior from those of ejaculation, we created three treatments: (1) “Naïve”: control males held in isolation from females. These males had no reproductive effort in the form of either mating behavior or ejaculation; (2) “Mating Only”: ablated males that could mate freely with females, but not ejaculate, and therefore did not invest in ejaculate replenishment; (3) “Mating *and* Ejaculation”: control males that could mate freely with females, and release sperm. Males were placed in their respective treatment for eight weeks, equivalent to two cycles of spermatogenesis (Koya and Iwase 2004) and approximately half of the length of the breeding season in the source population (Kahn et al. 2013). We then tested whether two key life history traits were affected by the treatments, namely, body growth and immune function. We also tested for a treatment effect on several measures of future reproductive performance: male attractiveness, male mate choice of larger females, male mating behavior, and sperm traits. By comparing the “naïve” and “mating only” treatments, we were able to quantify the costs of mating effort, and by comparing the “mating only” and “mating and ejaculation” treatments, we could quantify the costs of ejaculation that should accumulate with each copulation. Our experimental approach provides a unique insight into the relative cumulative costs of pre‐ and postcopulatory investments.

## Material and Methods

### FISH ORIGIN, MAINTENANCE, AND EXPERIMENTAL OVERVIEW

In March 2019, juvenile and adult *G. holbrooki* were collected in Canberra, Australia and housed in aquarium facilities at The Australian National University (under ANU Animal Ethics permit A2018/27). As soon as juveniles reached maturity (fully developed gonopodium with visible claws for males; a visible gravid spot for females), they were segregated into single‐sex tanks (40 fish per 90 L) to ensure virginity. Aquaria were maintained under a 14 L:10 D cycle at 28 ± 1°C. Fish were fed twice daily on commercial fish flake and *Artemia nauplii*.

**Figure 1:**
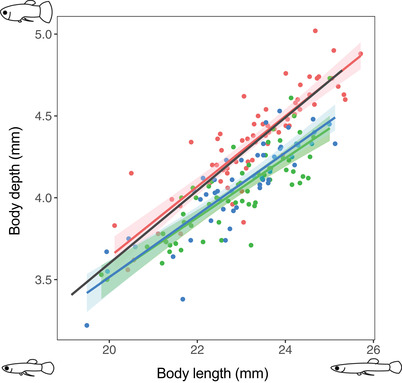
Effect of investment in pre‐ and postcopulatory components of reproduction by male *G. holbrooki* on somatic growth. Pre‐treatment body dimensions of all experimental males are represented by the black regression line. Post‐treatment body dimensions of males experiencing the three treatments: naïve (red); mating only (green); mating *and*ejaculation (blue) are shown along with regression lines and respective 95% confidence intervals.

Virgin males were first screened to ensure they were sexually active (defined as males that attempted to copulate with a wild‐caught female within 5 min of being in the same tank). These males were then anesthetized and photographed to measure their standard length (SL: snout tip to base of caudal fin) and body depth (BD: base of dorsal fin to low edge of body) using *ImageJ* (Abràmoff et al. 2004). While under anesthetic, each male underwent either gonopodium tip ablation or sham ablation (see Chung et al. 2019, 2020 for details). Size‐matched males (ANOVA, SL: *F*_2,174_ = 0.067, *P* = 0.936; BD: *F*_2,174_ = 0.331, *P* = 0.718; Fig. 1) were then haphazardly assigned into three treatments: (1) naïve: an intact male separated from a female by a mesh barrier that allowed for visual and olfactory stimuli, but prevented copulation (*n* = 66); (2) mating only: an ablated male housed with a free‐swimming female whom he could harass and mate with, but he could not ejaculate (*n* = 60); (3) mating *and* ejaculation: an intact male housed with a female whom he could harass, mate with, and inseminate (*n* = 51). All focal males were then housed alone in individual 4‐L tanks for 3‐day recovery, after which we introduced a wild‐caught female to each tank. Males were held in their respective treatments for eight weeks, during which time no regrowth of the gonopodium tip occured (personal observation). Females were rotated between tanks weekly to maintain male mating interest in novel females. At the end of the treatments, we collected data from males on two life history traits (growth and immunocompetence) and on four aspects of reproductive performance (male attractiveness, male mate choice for larger females, mating behavior, and sperm traits).

### PRELIMINARY TEST: EFFECT OF GONOPODIUM ABLATION ON MALE ATTRACTIVENESS

We randomly assigned size‐matched wild‐caught males (paired *t*‐test, SL: *t*_48_ = −0.247, *P* = 0.806) into an ablated or sham‐ablated (control) group (*n* = 49 male dyads). After a 3‐day recovery, we ran a two‐choice trial to quantify any female preference for associating with ablated versus intact, control males. We used a rectangular aquarium (40 × 23 × 10 cm) with two end compartments (5 × 23 × 10 cm) separated by mesh and an opaque screen from the middle section. An ablated and an intact male were randomly placed at opposite ends of the tank. A focal wild‐caught female that has been isolated from males for at least four weeks (28.43 ± 0.23 mm SL; *n* = 49) was placed in a Perspex cylinder in the middle of the tank for 10 min to acclimate. We then removed the opaque screens, lifted the Plexiglas cylinder, and videoed the female for 10 min to measure the time she spent in the association zone of each male (<5 cm from the mesh) and her total distance swum (Canon PowerShot G7X Mark II video camera). Videos were analyzed using Ethovision XT software (Noldus Information Technology, The Netherlands). Because female mating status does not influence her mate choice in *G. holbrooki* (Aich et al. 2020), we defined male attractiveness as the proportion of the total trial time the female spent within each association zone.

### DATA COLLECTION: COSTS OF REPRODUCTION MEASURED IN KEY LIFE HISTORY TRAITS

#### Body growth

At the end of the eight‐week experiment, males were rephotographed under anesthesia and measured via *ImageJ*. We then tested for treatment effects on growth by comparing males’ pre‐ and post‐treatment standard length and body depth.

#### Immune response

We tested the cell‐mediated immunity of males using a phytohemagglutinin (PHA) injection assay (verified in *G. holbrooki* and other fish species; Iglesias‐Carrasco et al. 2019; Petitjean et al. 2021). PHA‐stimulated response provides an effective evaluation of inflammation and cellular immune function (Goto et al. 1978), which has been linked to investments into pre‐ and postcopulatory traits (Velando et al. 2001; Simmons and Roberts 2005). With the male under anesthesia, we measured his body thickness at the posterior end of the dorsal fin with a pressure‐sensitive spessimeter (Mitutoyo 547‐301, accuracy: 0.01 mm), using the mean of five consecutive measures. We then injected a fixed volume of PHA (dissolved in PBS at 0.01 mg:0.01 ml) into the left side of the fish at the point where body thickness had been quantified. After 24 h, the body thickness was remeasured and the difference between the pre‐ and post‐injection values defined the individual's immune response.

### DATA COLLECTION: COSTS OF REPRODUCTION MEASURED AS FUTURE REPRODUCTIVE PERFORMANCE

#### Male attractiveness

We ran four‐choice trials to test for the cumulative effects of individual components of reproductive investment on male attractiveness. We used an aquarium (34 × 34 × 8 cm) with a compartment in each corner comprising a mesh divider and removable opaque screen. Three males (one per treatment) whose social treatments ended within ±1 day of each other were tested (*n* = 48 blocks). We haphazardly placed one male in each corner compartment, leaving the fourth compartment empty. A virgin female (28.45 ± 0.27 mm SL; *n* = 48) was introduced into a transparent Plexiglas cylinder in the center of the aquarium, with the males initially hidden behind opaque screens. Following 10‐min acclimation, we removed the screens and the holding cylinder. All females swum actively, and we recorded the time the female spent within each association zone for 10 min. Videos were analyzed using Ethovision XT. We interpreted the relative time spent with each male as his attractiveness.

#### Male mate choice

Cumulative investment in reproduction may affect male mate choice, either by enhancing the ability to assess female quality through experience (Iglesias‐Carrasco et al. 2019), or by biasing male mate choice toward lower quality females if such males have less to invest in reproduction (Holveck and Riebel 2010). We therefore tested whether past investment into individual reproductive components differentially affected a male's ability to discriminate between potential mates in two‐choice trials (see above). During the acclimatization phase, we randomly introduced a large (33.16 ± 0.18 mm SL) and a small female (25.42 ± 0.15 mm SL) into one of the end compartments (*n* = 69 female pairs), and each focal male was placed in a Perspex cylinder prior to release (see above). We defined male choosiness as the relative time he spent with the larger female. Pairs of females were used up to three times, but only once per male treatment. The order of testing was randomized.

#### Male mating behavior and the ability to obtain copulations

To test for the cumulative effect of investment in each reproductive component on a male's subsequent mating behavior, each male was introduced to a 4‐L aquarium with a novel virgin female (28.24 ± 0.11 mm SL; *n* = 176) behind a mesh screen. After a 10‐min acclimation period, we raised the barrier and for 20 min recorded (a) the number of copulatory attempts; (b) time spent within one SL of the female; and (c) total distance swum. Copulatory attempts were counted directly, and the other two measures were obtained from video recordings using Ethovision XT.

#### Sperm traits

We evaluated the effects of investment in different reproductive components on (a) sperm count, (b) rate of sperm replenishment, and (c) sperm velocity. We first stripped sperm reserves of each male at the end of the eight‐week treatment (Day 0) to standardize sperm age and time since ejaculation (Gasparini et al. 2019). Males were returned to individual tanks and housed alone for five days to replenish sperm reserves (O'Dea et al. 2014). On Day 5, we re‐stripped males to obtain an ejaculate sample to measure sperm count and velocity. After 24 h, males were stripped again to quantify the sperm replenishment rate. The method of sperm collection followed that outlined in Gasparini et al. (2013) and Vega‐Trejo et al. (2019). Sperm parameters were captured using Computer‐Assisted Sperm Analysis (CEROS Sperm Tracker, Hamilton Thorne, USA), see Supporting Information for details. Sperm counts for each male were based on the mean of five subsamples counted within a 3‐μl sample on a 20‐micron capillary slide (Leja) (repeatability: *r* ± SE = 0.904 ± 0.008, *P* < 0.001, *n* = 339 male‐days). Sperm velocity was calculated as the average curvilinear velocity (VCL) of all motile tracks with two samples per male (55.1 ± 2.4 SE sperm tracks per ejaculate).

### STATISTICAL ANALYSES

To test for any effect of gonopodium ablation on male attractiveness, we used a generalized linear mixed model (GLMM) with negative binomial error and time spent with each male as the response variable. We considered the state of gonopodium as a fixed factor, female identity as a random factor, and male size difference was included as a covariate to control for potential differences in male attractiveness (although in all but a handful of cases the difference in body size between males was <1 mm). We also included the size difference*gonopodium state interaction in the model.

In our main experiments, we ran separate general linear models to test for a treatment effect on (a) body growth, (b) immune response, (c) distance swum and total time spent near each of the females in two‐choice trials, (d) distance swum and time spent following the female in mating‐behavior trials, and (e) sperm traits (count, velocity, replenishment rate). Separate generalized linear models were used to investigate the relative time spent associating with a larger female (quasi‐Poisson error) and the number of copulatory attempts (negative binomial error). To examine any differences in male attractiveness, we used a two‐step analysis. We first tested whether females spent more time with males than was expected by chance: we ran a one‐sample *t*‐test to evaluate whether the time spent in the empty corner divided by total time in the four association zones was less than 0.25. The total time a female spent with males was 62 ± 3% of the trial. We then tested for a female preference for the three types of males using a GLMM (negative binomial error). We treated the association time with each male as the dependent variable and female identity as a random factor. In all main experiment models, treatment was a fixed factor, standardized initial body size was a covariate, and their interaction was included. For the analysis of two‐choice trials, we also considered female size difference as a covariate, as a greater value might enhance the male's ability to identify the larger female. For all models, if the treatment effect was significant, we ran Tukey's post hoc tests to examine pairwise differences between groups.

For all analyses, nonsignificant interactions were excluded without changing the best fit of the model (see Supporting Information). This allowed us to examine the main treatment effect. Results are presented as mean ± SE, and considered significant if *P* ≤ 0.05 (two‐tailed). Sperm count data were log‐transformed to fulfill model assumptions. Males with values >3 SDs from the mean of their treatment were excluded: one for the number of mating attempts, one for the total distance swum of mating behavior, two for total sperm count, three for sperm replenishment rate, and one for sperm velocity. One choice trial was excluded because the female did not spent time in either association zone. Results including these outliers are presented in the Supporting Information. Our analyses were planned and preregistered online (https://osf.io/78jht).

## Results

### ABLATION EFFECT ON MALE ATTRACTIVENESS

Controlling for size difference in each male pair, females did not discriminate ablated males as a result of the shape of his gonopodium tip (*χ* ^2^_1,91_ = 0.001; *P* = 0.973), indicating that ablation did not increase the effort males would need to make in order to associate with females for the purposes of mating. We therefore attributed all subsequent phenotypic differences among the treatments to the longer‐term costs of pre‐ and/or postcopulatory investment.

### COSTS OF INDIVIDUAL REPRODUCTIVE COMPONENTS MEASURED IN KEY LIFE HISTORY TRAITS

**Figure 2:**
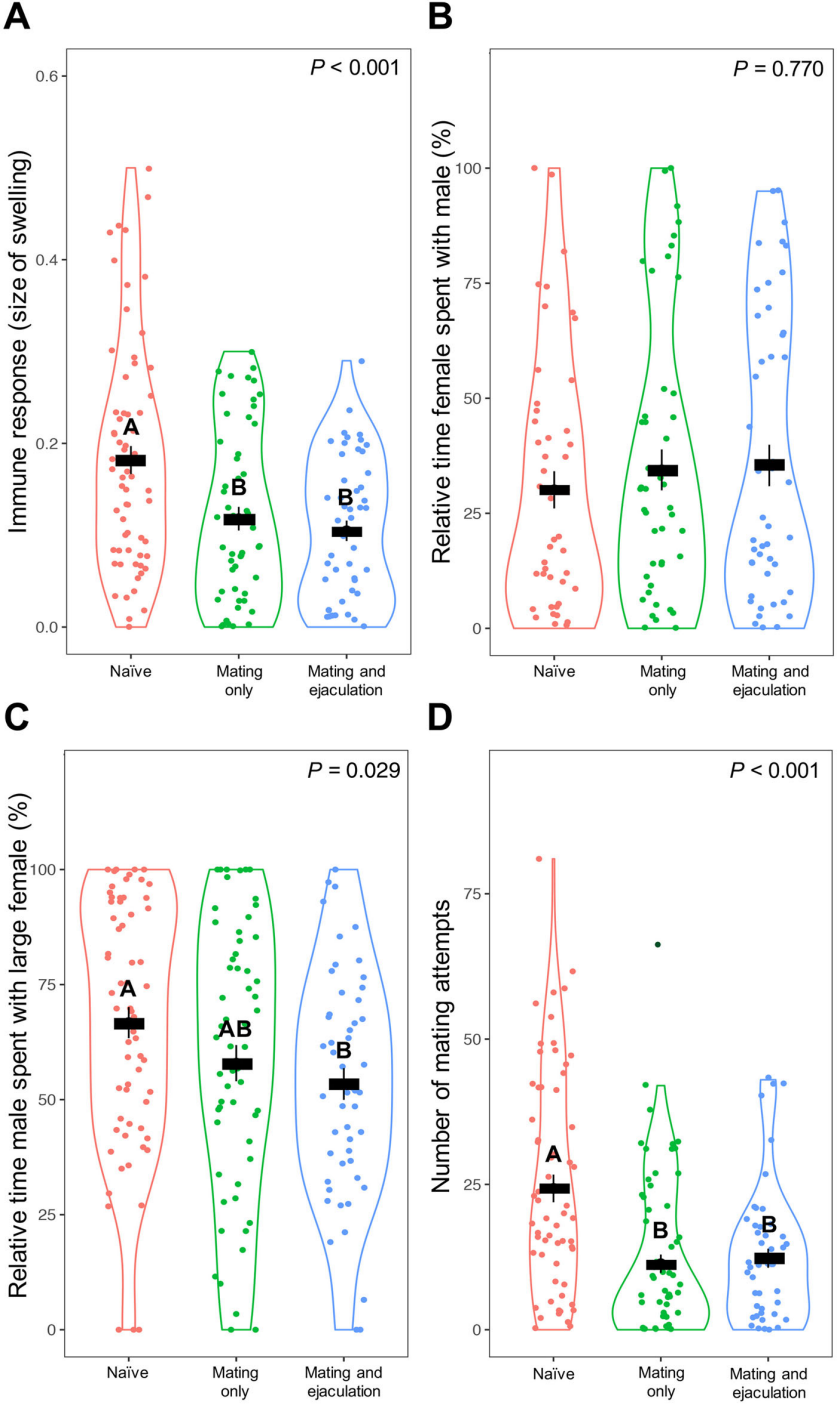
Violin plots showing the effect of investment in pre‐ and postcopulatory components of reproduction by male *G. holbrooki* on (A) immune response, (B) male attractiveness, (C) male mate choice (preference for the larger of two females), and (D) number of mating attempts. Colors represent the three treatments: naïve (red); mating only (green); mating *and* ejaculation (blue). One male outlier (dark green point in [D]) was excluded from the statistical analyses. Letters represent significant differences among treatments based on Tukey's tests. Black bars represent mean ± *SE*

Male mating effort (i.e., female harassment and mating itself) had a significant effect on male growth (Table 1; Fig. 1). Controlling for initial body size, the post‐treatment SL and BD of the naïve males were significantly greater than that of the ablated, “mating only” males that could harass and mate with females, but not ejaculate (Tukey's test, SL: *P* < 0.001; BD: *P* < 0.001). Interestingly, however, ejaculation and subsequent sperm replenishment by males did not result in any *additional* decline in growth (Fig. 1; Tukey's test, SL: *P* = 0.998; BD: *P* = 0.983). Male mating effort also had a significant effect on their immune function (Table 1). Compared to naïve males that incurred no mating costs, the “mating only” ablated males had significantly lower immunocompetence (Fig. 2A; Tukey's test, *P* = 0.002). Again, however, ejaculation by intact males had no additional negative effects on immune function, compared to those observed for ablated males (Fig. 2A; Tukey's test, *P* = 0.810).

**Table 1:** Effect of level of investment in pre‐ and postcopulatory components of reproduction by male *G. holbrooki* (naïve, mating only, mating *and* ejaculation) on (A) life history traits and (B) reproductive performance traits of males

Trait	Predictor	Test statistic	*P*‐value
(a) Life history traits			
(i) *Body growth*			
Body length (SL)	Level of mating costs	*F*_2,173_ = 14.852	<0.001
Body depth (BD)	Level of mating costs	*F*_2,173_ = 71.058	<0.001
(ii) *Immune response*	Level of mating costs	*F*_2,167_ = 9.582	<0.001
(b) Post‐treatment reproductive performance traits (potential late‐life trade‐offs)			
Precopulatory traits			
(iii) *Male attractiveness*	Level of mating costs	*χ* ^2^_2,138_ = 0.522	0.770
(iv) *Male mate choice*			
Time with large female	Level of mating costs	*χ* ^2^_2,170_ = 7.073	0.029
Total distance swum	Level of mating costs	*F*_2,170_ = 1.109	0.332
Total inspection time	Level of mating costs	*F*_2,170_ = 2.910	0.057
(v) *Male mating behavior*			
Number of mating attempts	Level of mating costs	*χ* ^2^_2,171_ = 16.594	<0.001
Total distance swum	Level of mating costs	*F*_2,169_ = 0.003	0.998
Time spent with female	Level of mating costs	*F*_2,170_ = 0.746	0.476
Postcopulatory traits			
(vi) *Sperm count*	Level of mating costs	*F*_2,163_ = 13.171	<0.001
	Level of costs* male size	*F*_2,163_ = 6.104	0.003
(vii) *Sperm replenishment rate*	Level of mating costs	*F*_2,159_ = 3.183	0.044
	Level of costs* male size	*F*_2,159_ = 3.307	0.039
(viii) *Sperm velocity (VCL)*	Level of mating costs	*F*_2,167_ = 0.365	0.695
	Level of costs* male size	*F*_2,165_ = 0.422	0.657

* Detailed statistical analyses of each trait with and without outliers are provided in Supporting Information.

### COSTS OF INDIVIDUAL REPRODUCTIVE COMPONENTS MEASURED AS FUTURE REPRODUCTIVE PERFORMANCE

Males’ cumulative investment into reproductive effort had no effect on their attractiveness. In four‐choice trials, females preferred to associate with males rather than the empty corner (*t*_47_ = 4.228, *P <* 0.001), but they did not discriminate among males from the three treatments (Table 1; Fig. 2B). There was a significant cumulative effect of the level of mating investment on male mate choice, but only when comparing naïve males with those that had invested in both mating *and* ejaculation (Table 1; Fig. 2C; Tukey's test, *P* = 0.028). Naïve males and males that only mated spent significantly more than 50% of their inspection time associating with the larger of two females (naïve males [*t*_63_ = 4.986, *P* < 0.001], “mating only” males [*t*_59_ = 2.016, *P* = 0.048]), whereas males that had both mated and released sperm showed no such preference (*t*_50_ = 0.987, *P* = 0.328) (all one‐sample *t*‐tests). There were no differences among the three treatments in the overall time spent with the females or distance swum during the trial (Table 1).

We found no treatment effect on subsequent male mating activity, namely, time spent following the female or the distance swum (Table 1). In contrast, males that had cumulatively invested in mating effort over the eight‐week period performed significantly fewer copulation attempts than naïve males during subsequent mating trials (Table 1; Fig. 2D; Tukey's test, *P* < 0.001). There was, however, no additional effect of cumulative investment in ejaculation and subsequent sperm replenishment (Tukey's test, *P* = 0.929).

**Figure 3:**
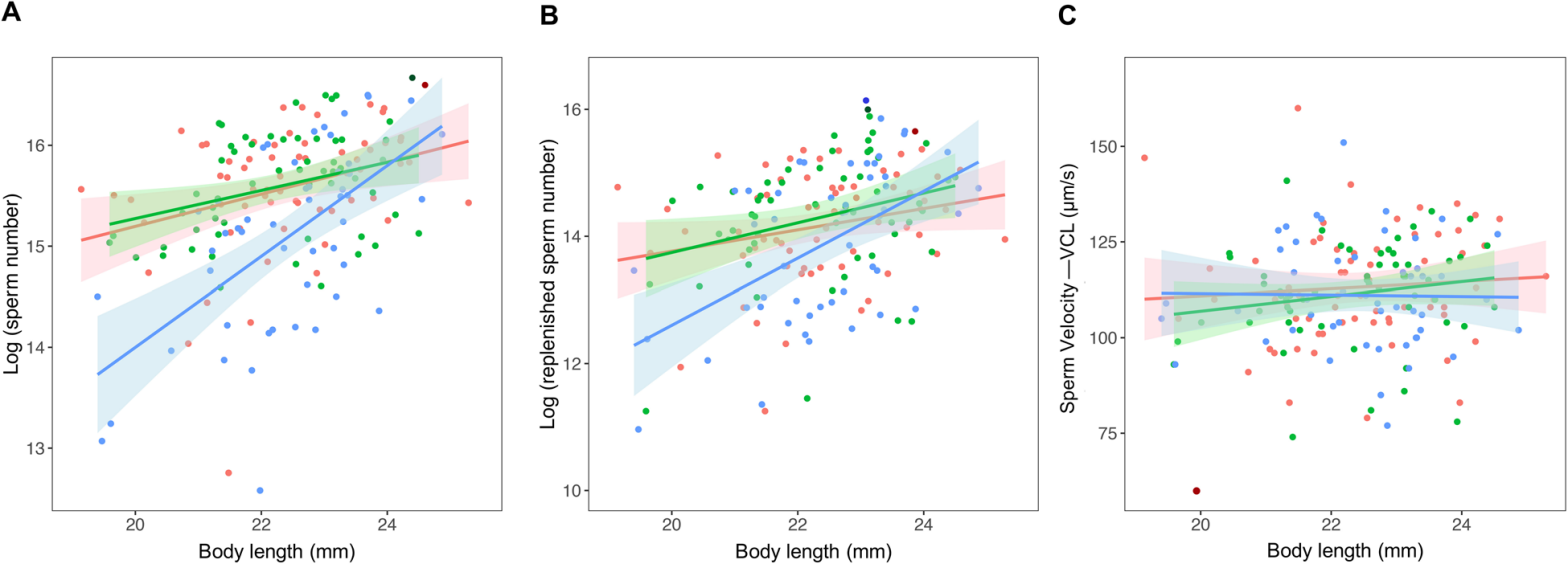
The effects of cumulative investment into mating effort (either with or without ejaculation) on the sperm traits of *G. holbrooki*, controlling for male body size. Effect of the three mating treatments (no investment in mating by naïve males [red], investment in mating only [green], or mating *and* ejaculation [blue]) on (A) total sperm count, (B) rate of sperm replenishment, and (C) sperm velocity. The sample distribution, regression line, and its 95% confidence interval for the three treatments are shown. Points with darker colors represent outliers excluded from the statistical analyses.

Cumulative investment into mating effort by males had no effect on sperm traits (Table 1; Fig. 3). Intriguingly, although there was an effect of cumulative investment in ejaculation on a male's subsequent sperm count and rate of sperm replenishment, this was only the case for smaller individuals (significant interaction between level of mating investment and male size; Table 1). Small males that had invested in mating *and* ejaculation had lower sperm counts (Fig. 3A) and slower sperm replenishment rates (Fig. 3B) than naïve and “mating only” counterparts. Neither mating effort nor ejaculate release had a significant effect on subsequent sperm velocity (Table 1; Fig. 3C).

## Discussion

In this study, we used a novel morphological manipulation to tease apart the cumulative costs of mating effort and ejaculation for *G. holbrooki*, providing a unique insight into the relative costs of each component of male reproduction. An early study in *Drosophila melanogaster* used microcautery to seal the female vagina to demonstrate the contribution of courtship and sperm production to male longevity (Cordts and Partridge 1996), but our study is, to our knowledge, the first to experimentally separate the costs of mating from those of ejaculation in a vertebrate. Our results show that acquiring mates and mating are costly for males due to their significant negative effect on key life history traits, namely, growth and immune function. Mating behavior also incurs a future sexually selected cost because mating effort seemingly lowers future reproductive success by reducing the rate at which males attempt to mate. Surprisingly, however, there was no detectable cost of ejaculation (and subsequent sperm replenishment) on life history traits despite the fact that *G. holbrooki* is a species with intense sperm competition that favors high investment into ejaculate traits (Evans et al. 2003; Lüpold et al. 2020). The only effects detected were (a) a decrease in sperm count and sperm replenishment rate, but this was only for smaller males, and (b) a decline in a male's preference for larger, more fecund, females. The observed low somatic cost of ejaculation in *G. holbrooki* is likely to be seen in many other vertebrates because the high frequency with which mosquitofish males mate (Wilson 2005) elevates the likelihood of detecting costs of ejaculation.

The finding that male reproductive effort trades off against growth in *G. holbrooki* echoes similar findings in guppies (Miller and Brook 2005) and other taxa, including insects (Himuro and Fujisaki 2010), reptiles (Schwarzkopf 1993; Olsson et al. 1997), amphibians (Given 1988), and mammals (Michener and Locklear 1990; Ryser 1992). Similarly, the suppression of immune function by males that invested more into reproduction is consistent with findings from zebra finches (Deerenberg et al. 1997), great tits (Richner et al. 1995), and a recent study of *G. holbrooki* (Iglesias‐Carrasco et al. 2019). Although a somatic cost of reproduction is taxonomically widespread, the underlying causal mechanisms seem to vary. For example, the cost is partly due to reduced foraging time and lower food intake in guppies (Griffiths 1996), the depletion of energetic reserves when courting in field crickets (Hunt et al. 2004), and the immunomodulatory effects of testosterone in red‐legged partridge (Blas et al. 2006; meta‐analysis: Foo et al. 2017). This variation begs the question of the extent to which these costs are due to investment into mating effort versus ejaculate replenishment. Different forms of investment might impose different costs.

In most studies, the relative contribution of investment into traits under pre‐ versus postcopulatory sexual selection on the costs of reproduction for males is not distinguishable (Dao et al. 2010; Himuro and Fujisaki 2010; Metzler et al. 2016; Iglesias‐Carrasco et al. 2019): males with a higher mating effort copulate more often, and therefore have to ejaculate and replenish their sperm supplies more often. In addition, many studies are correlational and natural variation in resource acquisition among males can easily obscure the costs of greater investment (Reznick et al. 2000). This is why experiments are required to identify the costs of reproduction. In our study, we took advantage of a novel surgical manipulation (intromittent organ tip ablation) to distinguish the costs of mating effort and ejaculation in *G. holbrooki*. In so doing, we found that mating effort *alone* imposes significant somatic costs on males. One possible reason is that mating activity elevates testosterone levels (Toft et al. 2003; Toft and Guillette 2005), which has been shown to inhibit growth and immunocompetence in other vertebrates (review: Cox 2020). Another explanation is that, due to our study design, males in two of the three treatments had to compete for food with females, although we minimized variation in food availability among test males by providing excess *A. nauplii*. In contrast, investment in ejaculation had no detectable cumulative somatic costs for male *G. holbrooki*.

Our finding of no detectable somatic costs of ejaculation seems at odds with general claims that sperm production is costly (Devigili et al. 2017; review: Simmons et al. 2017). It is not without precedent, however, as an experiment with *D. melanogaster* also showed that sperm production does not lower male longevity over and above that attributable to courtship and mating (Cordts and Partridge 1996). A range of lines of evidence do, however, exist within the literature to suggest that sperm production is costly: first, the fact that ejaculate traits are sensitive to diet (Devigili et al. 2013; Rahman et al. 2013; Kahrl and Cox 2015; Kaldun and Otti 2016) and to the presence of particular nutrients (Rahman et al. 2014a,b; Dávila and Aron 2017; review: Macartney et al. 2019). Second, sperm depletion suggests that there are limits to the ability to produce sperm at a high rate (Hughes et al. 2000; Preston et al. 2001). Finally, strategic allocation of ejaculates in response to social cues (Wedell et al. 2002; Kelly and Jennions 2011; Cattelan and Pilastro 2018; Cardozo et al. 2020) also indicates that ejaculates are a limited resource, consistent with their production being costly. However, we recently showed that male *G. holbrooki* have a lower risk of sperm depletion and do not adjust ejaculate components in response to social status or female cues (Chung et al. 2019). Even though food availability affects the number of sperm produced by *G. holbrooki* (O'Dea et al. 2014), such an effect does not necessarily lead to long‐term costs. For example, in some species males might compensate for a greater demand of ejaculate release by adjusting their food intake (Soulsbury 2019). In species that do not feed during the mating period, such as adders (Olsson et al. 1997), an effect of ejaculate production on somatic traits might be more likely to be observed because males have to allocate fixed resources between maintenance and spermatogenesis (i.e., capital breeders; Doughty and Shine 1997). In *G. holbrooki*, males continue to forage while breeding, so it is plausible that they might simply increase their rate of feeding to compensate for the immediate costs of ejaculation, resulting in no decline in somatic condition. Even so, a compensatory elevation in foraging effort is likely to have some negative long‐term effect on somatic traits. Ultimately, it seems inevitable that there are some costs to replenishing sperm from ejaculation but our failure to detect them suggests that these costs may be only imposed very late in life (i.e., a reduction in lifespan or faster senescence, neither of which we measured), and/or that the costs themselves are very small (e.g., low foraging costs under laboratory conditions). In addition, we acknowledge that although naïve and ablated males might continue to produce sperm by re‐absorbing or discharging unused sperm, the rate of replacing older, stored sperm will still be lower than that due to replenishing sperm after ejaculation. This is consistent with evidence that unmated males have older sperm (Gasparini et al. 2014, 2019). More importantly, any such mechanism of renewing sperm reserves would not affect the extent to which intact males incur an additional cost because of ejaculation during copulation.

Our results failed to show the trade‐off between pre‐ and postcopulatory traits that has been reported in many studies (Parker 1998; Simmons and Emlen 2006; Lüpold et al. 2014; Simmons et al. 2017). That is, males who invested in ejaculation showed no subsequent elevated decline in mating effort, at least over the eight‐week test period. Instead, there was evidence of a trade‐off between current and future performance within each type of trait: greater mating effort reduced the subsequent rate at which males made mating attempts, and greater frequency of ejaculation decreased future sperm count and sperm replenishment rate. Our results clearly demonstrate that “mating only” and “mating *and* ejaculation” males invested similarly in mating activity throughout the experimental period (i.e., from the start [Chung et al. 2020] to the end of the treatments [Fig. 2D]), in line with our previous findings of no surgery costs for ablated males (Chung et al. 2019; Fox et al. 2019). When compared with the naive males, this provides evidence that past mating effort alone can lower future investment into mating. One explanation is that male mating effort is condition‐dependent and early investment into acquiring copulations leads to a decline in somatic state due to lower immunocompetence and smaller energy reserves (as implied by lower growth), then making it more costly for males to pursue females. A similar argument can be made for sperm production when it is condition‐dependent (O'Dea et al. 2014). Of course, we cannot rule out the possibility that trade‐offs between pre‐ and postcopulatory traits become more evident over an even longer period than eight weeks, and we will investigate this possibility in a future study.

Intriguingly, in our current study ejaculation costs seemed to be size‐dependent: only smaller males showed lower sperm production after being in the mating and ejaculation treatment compared to the other two treatments (Fig. 3). This is consistent with smaller males having a lower capacity for energy storage given the costs of reproduction (e.g., Pitnick and Markow 1994). In contrast, after eight weeks there was a decrease in mating effort in males of all sizes in response to past mating effort (i.e., comparing the naïve males with the other two treatments) (Fig. 2D). Our results show some similarity with the more pronounced senescence of male precopulatory traits than sperm traits shown in guppies (Gasparini et al. 2019). It remains unclear why there was only lower ejaculate production in the “mating and ejaculation” treatment of our study. This finding suggests that there is direct damage to tissue and structures uniquely associated with ejaculate production, such as long‐term damage to testes. It is also unclear why mating effort alone did not lead to a decline in sperm production if investment in mating decreases the resources available for sperm investment. It might simply be that the cost of mating effort arises due to repeated physical behavior causing the accumulation of oxidative stress (Powers and Jackson 2008) or extra damage to particular muscles or tissues (e.g., tearing of fins during high‐speed swimming when harassing the female) other than those involved in sperm production.

In sum, by using a novel ablation technique in mosquitofish, we quantified the relative costs of mating effort and ejaculation. Our finding that the cumulative somatic costs of ejaculation are barely detectable compared to those of mating behavior is intriguing given the multiple lines of evidence from other taxa that sperm production is costly (Wedell et al. 2002; Simmons et al. 2017). It suggests that any immediate costs of ejaculation might be offset by high food availability in the laboratory (e.g., Cordero 2000; Landes et al. 2019). An interesting next step would be to test whether more stressful conditions (e.g., low food availability), or even an environment that favors still higher sperm production (e.g. higher risk of sperm competition due to the presence of rivals), could increase the net costs of ejaculate investment so that they become detectable. If so, this could potentially alter energy allocation into pre‐ and postcopulatory traits such that the observed trade‐offs are no longer only within each sexual trait type as detected in our current study.

## AUTHOR CONTRIBUTIONS

MHJC, MDJ, and RJF conceived and designed the project. MHJC collected and analyzed the data and wrote the first draft. All authors contributed to, and approved, the final manuscript. The authors are accountable for the work performed therein.

## DATA ARCHIVING

Raw data are provided as a separate file in the Supporting Information.

## CONFLICT OF INTEREST

The authors declare no conflict of interest.

## Supporting information

Figure 1. Upper views of experimental apparatus for (A) two‐choice trials and (B) four‐choice trials. A focal fish was housed at the center of the tank within a plastic cylinder for 10 min and then released to swim freely around the mid‐ and association sections. We treated the time spent in each association zone as mating preference. A, mid‐section of tank; B, association zone; C, end section containing stimulus fish; D, mesh barrier and mobile opaque screen; E, plastic container; F, black barrier.

Table S1. Measurements of male attractiveness after 3‐day recovery from ablating the gonopodium tip
Table S2. Measurements of survival‐related somatic traits and reproductive performance traits after males underwent different levels of mating costs.
Table S3. Measurements of male attractiveness after males underwent different levels of mating costs.
