# Supplementary material for: Quantifying the costs of pre‐ and postcopulatory traits for males: Evidence that costs of ejaculation are minor relative to mating effort
Source: Evol Lett. 2021 May 27;5(4):315–27. doi: 10.1002/evl3.228 (PMC8327938; doi:10.1002/evl3.228)
Supplement: Supplementary file 1 — Figure 1. Upper views of experimental apparatus for (A) two‐choice trials and (B) four‐choice trials. A focal fish was housed at the center of the tank within a plastic cylinder for 10 min and then released to swim freely around the mid‐ and association sections. We treated the time spent in each association zone as mating preference. A, mid‐section of tank; B, association zone; C, end section containing stimulus fish; D, mesh barrier and mobile opaque screen; E, plastic container; F, black barrier. [file EVL3-5-315-s002.docx]

Supporting Information

**Quantifying the costs of pre- and post-copulatory traits for males: evidence that costs of ejaculation are minor relative to mating effort**

Meng-Han Joseph Chung^1^, Michael D. Jennions^1^, Rebecca J. Fox^1^

1. Division of Ecology and Evolution, Research School of Biology, Australian National University, Canberra, ACT, Australia

**Author for correspondence:**

Meng-Han Joseph Chung, e-mail: [meng-han.chung@anu.edu.au](mailto:meng-han.chung@anu.edu.au)

***Part 1. Tank designs for two-choice and four-choice mating trials***

**Figure 1**. Upper views of experimental apparatus for (A) two-choice trials and (B) four-choice trials. A focal fish was housed at the center of the tank within a plastic cylinder for 10 min and then released to swim freely around the mid- and association sections. We treated the time spent in each association zone as mating preference. A, mid‑section of tank; B, association zone; C, end section containing stimulus fish; D, mesh barrier and mobile opaque screen; E, plastic container; F, black barrier.

***Part 2. Experimental protocols for sperm traits***

When males were anaesthetised, we placed them on a glass slide coated in 1% polyvinyl alcohol solution (PVA), swung the gonopodium forward and gentle stripped the abdomen with a probe to stimulate ejaculation. We immediately pipetted 100μl of extender medium (207 mM NaCl, 5.4 mM KCl, 1.3 mM CaCl_2_, 0.49 mM MgCl_2_, 0.41 mM MgSO_4_, 10 mM Tris(Cl); pH 7.5) onto the slide to hydrate the sample. Under a dissecting microscope we then collected and transferred two samples of 3 individual sperm bundles each to an Eppendorf tube containing 2μl of extender medium from which to measure sperm velocity. We collected the remaining ejaculate into a 1.5ml Eppendorf tube containing a known volume (500-1000μl) of extender medium to estimate the sperm count.

**Sperm count**

We vortexed the sperm solution (into a 1.5ml Eppendorf tube) for 30 sec and used a 10μl pipette to break up sperm bundles. We placed 3μl on a 20-micron capillary slide (Leja) to calculate the sperm number using the program CEROS Sperm Tracker (Hamilton Thorne Research, USA) under 100× magnification. We calculated the mean from five subsamples per sample.

**Sperm velocity**

We evaluated sperm velocity from two samples per male as the average of all the sperm tracks recorded. For each sample, we pipetted the 3μl solution of sperm bundles and extender medium into the centre of a cell in a 12‐cell multi-test slide (MP Biomedicals, USA) covered with 1% PVA. Each sample was activated with 3μl of 125 mM KCl and 2 mg/ml bovine serum albumin (Billard and Cosson 1992) for 30 sec and then covered with a coverslip. We measured the velocity for 55.1 ± 2.4 *SE* sperm tracks per ejaculate and recorded (a) average path velocity (VAP): the average velocity over a smoothed cell path and (b) curvilinear velocity (VCL): the actual velocity along the trajectory, and (c) straight-line velocity (VSL) using the CEROS Sperm Tracker (Hamilton Thorne Research). The threshold values defining static cells were set at 20 μm/sec for VAP and 15 μm/sec for VSL (Gasparini et al. 2010). Given that VAP, VSL and VCL are highly correlated in the mosquitofish (Iglesias‐Carrasco et al. 2020), we therefore reported the more biologically relevant VCL, which has been shown to positively correlate with male fertilisation success in a closely related poeciliid species (Boschetto et al. 2011).

**References**

Billard, R. & Cosson, M.P. (1992). Some problems related to the assessment of sperm motility in freshwater fish. *J. Exp. Zool.* 261:122–131.

Boschetto, C., Gasparini, C. & Pilastro, A. (2011). Sperm number and velocity affect sperm competition success in the guppy (*Poecilia reticulata*). *Behav. Ecol. Sociobiol.* 65:813–821.

Gasparini, C., Marino, I.A.M., Boschetto, C. & Pilastro, A. (2010). Effect of male age on sperm traits and sperm competition success in the guppy (*Poecilia reticulata*). *J. Evol. Biol.* 23:124–135.

Iglesias‐Carrasco, M., Harrison, L., Jennions, M.D. & Head, M.L. (2020). Combined effects of rearing and testing temperatures on sperm traits. *J. Evol. Biol.* 33:1715–1724.

***Part 3. Model outputs from statistical analyses of effect of ablation and male size difference on male attractiveness***

(a) Effect of different states of the gonopodium tip with outlier included.

|  | df | χ² value | P value |
| --- | --- | --- | --- |
| Intercept | 1 | 1553.639 | <0.001 |
| Male size difference | 1 | 0.568 | 0.451 |
| Treatment | 1 | 0.001 | 0.976 |

(b) Exclusion of the interaction effect (gonopodium state*male size difference) did not significantly reduce model fit. Therefore, main effects reported from reduced model.

|  | df | AIC | BIC | Log-likelihood | Deviance | χ² value | χ² df | P value |
| --- | --- | --- | --- | --- | --- | --- | --- | --- |
| Model with the interaction effect | 6 | 1117.1 | 1132.5 | -552.57 | 1105.1 | 0.183 | 1 | 0.669 |
| Model without the interaction effect | 5 | 1115.3 | 1128.1 | -552.66 | 1105.3 |  |  |  |

***Part 4. Model outputs from statistical analyses of effect of mating treatment and male body size on male somatic and reproductive traits***

**Trait 1. Male Growth**

- 1. **Increase in standard length (SL)**

(a) Effect of different levels of mating costs with outlier included

|  | df | Sum sq | F value | P value |
| --- | --- | --- | --- | --- |
| Intercept | 1 | 31517.2 | 223301.666 | <0.001 |
| Initial body length (standardised) | 1 | 248.2 | 1758.663 | <0.001 |
| Treatment | 2 | 4.2 | 14.852 | <0.001 |
| Residuals | 173 | 24.4 |  |  |

(b) Exclusion of the interaction effect (mating treatment*male body size) did not significantly reduce model fit. Therefore, main effects reported from reduced model.

|  | Res.df | RSS | df | Sum sq | F value | P value |
| --- | --- | --- | --- | --- | --- | --- |
| Model with the interaction effect | 171 | 24.183 |  |  |  |  |
| Model without the interaction effect | 173 | 24.418 | -2 | -0.234 | 0.829 | 0.438 |

- 1. **Increase in body depth (BD)**

(a) Effect of different levels of mating costs with outlier included

|  | df | Sum sq | F value | P value |
| --- | --- | --- | --- | --- |
| Intercept | 1 | 982.60 | 37432.950 | <0.001 |
| Initial body depth (standardised) | 1 | 9.86 | 375.593 | <0.001 |
| Treatment | 2 | 3.73 | 71.058 | <0.001 |
| Residuals | 173 | 4.54 |  |  |

(b) Exclusion of the interaction effect (mating treatment*male body size) did not significantly reduce model fit. Therefore, main effects reported from reduced model.

|  | Res.df | RSS | df | Sum sq | F value | P value |
| --- | --- | --- | --- | --- | --- | --- |
| Model with the interaction effect | 171 | 4.470 |  |  |  |  |
| Model without the interaction effect | 173 | 4.541 | -2 | -0.071 | 1.364 | 0.258 |

**Trait 2. Immune response**

(a) Effect of different levels of mating costs with outlier included

|  | df | Sum sq | F value | P value |
| --- | --- | --- | --- | --- |
| Intercept | 1 | 0.762 | 71.575 | <0.001 |
| Initial body length (standardised) | 1 | 0.011 | 1.078 | 0.301 |
| Treatment | 2 | 0.204 | 9.582 | <0.001 |
| Residuals | 167 | 1.777 |  |  |

(b) Exclusion of the interaction effect (mating treatment*male body size) did not significantly reduce model fit. Therefore, main effects reported from reduced model.

|  | Res.df | RSS | df | Sum sq | F value | P value |
| --- | --- | --- | --- | --- | --- | --- |
| Model with the interaction effect | 165 | 1.763 |  |  |  |  |
| Model without the interaction effect | 167 | 1.777 | -2 | -0.014 | 0.664 | 0.516 |

**Trait 3. Male attractiveness**

(a) Effect of different levels of mating costs with outlier included.

|  | df | χ² value | P value |
| --- | --- | --- | --- |
| Intercept | 1 | 704.209 | <0.001 |
| Initial body length (standardised) | 1 | 2.556 | 0.110 |
| Treatment | 2 | 0.522 | 0.770 |

(b) Exclusion of the interaction effect (mating treatment*male body size) did not significantly reduce model fit. Therefore, main effects reported from reduced model.

|  | df | AIC | BIC | Log-likelihood | Deviance | χ² value | χ² df | P value |
| --- | --- | --- | --- | --- | --- | --- | --- | --- |
| Model with the interaction effect | 8 | 1669.5 | 1693.2 | -826.73 | 1653.5 | 1.813 | 2 | 0.404 |
| Model without the interaction effect | 6 | 1667.3 | 1685.1 | -827.64 | 1655.3 |  |  |  |

**Trait 4. Male mate-choice**

**4-1 Time spent associating with large female**

(a) Effect of different levels of mating costs with outlier included

|  | df | χ² value | P value |
| --- | --- | --- | --- |
| Intercept | 1 | 8.192 | 0.004 |
| Female size difference | 1 | 0.168 | 0.682 |
| Initial body length (standardised) | 1 | 0.785 | 0.376 |
| Treatment | 2 | 7.073 | 0.029 |

(b) Exclusion of the interaction effect (mating treatment*male body size) did not significantly reduce model fit. Therefore, main effects reported from reduced model.

|  | Residual df | Residual deviance | df | Deviance |
| --- | --- | --- | --- | --- |
| Model with the interaction effect | 168 | 27.284 |  |  |
| Model without the interaction effect | 170 | 28.024 | -2 | -0.741 |

**4-2 Total distance swum**

(a) No effect of different levels of mating costs with outlier included.

|  | df | Sum sq | F value | P value |
| --- | --- | --- | --- | --- |
| Intercept | 1 | 5454001 | 21.202 | <0.001 |
| Female size difference | 1 | 456374 | 1.774 | 0.185 |
| Initial body length (standardised) | 1 | 312720 | 1.216 | 0.272 |
| Treatment | 2 | 570767 | 1.109 | 0.332 |
| Residuals | 170 | 43731209 |  |  |

(b) Exclusion of the interaction effect (mating treatment*male body size) did not significantly reduce model fit. Therefore, main effects reported from reduced model.

|  | Res.df | RSS | df | Sum sq | F value | P value |
| --- | --- | --- | --- | --- | --- | --- |
| Model with the interaction effect | 168 | 42268351 |  |  |  |  |
| Model without the interaction effect | 170 | 43731209 | -2 | -1462858 | 2.907 | 0.057 |

**4-3 Total inspection time**

(a) No effect of different levels of mating costs with outlier included.

|  | df | Sum sq | F value | P value |
| --- | --- | --- | --- | --- |
| Intercept | 1 | 307381 | 16.865 | <0.001 |
| Female size difference | 1 | 40976 | 2.248 | 0.136 |
| Initial body length (standardised) | 1 | 13586 | 0.745 | 0.389 |
| Treatment | 2 | 106081 | 2.910 | 0.057 |
| Residuals | 170 | 3098489 |  |  |

(b) Exclusion of the interaction effect (mating treatment*male body size) did not significantly reduce model fit. Therefore, main effects reported from reduced model.

|  | Res.df | RSS | df | Sum sq | F value | P value |
| --- | --- | --- | --- | --- | --- | --- |
| Model with the interaction effect | 168 | 3095224 |  |  |  |  |
| Model without the interaction effect | 170 | 3098489 | -2 | -3265.1 | 0.089 | 0.915 |

**Trait 5. Male mating behaviour**

**5-1 Number of mating attempts**

(a) Effect of different levels of mating costs (excluding one outlier)

|  | df | χ² value | P value |
| --- | --- | --- | --- |
| Intercept | 1 | 261.046 | <0.001 |
| Initial body length (standardised) | 1 | 0.012 | 0.912 |
| Treatment | 2 | 16.594 | <0.001 |

(b) Exclusion of the interaction effect (mating treatment*male body size) did not significantly reduce model fit. Therefore, main effects reported from reduced model.

|  | θ | Res.df | 2*Log-likelihood | df | LR statistic | P value |
| --- | --- | --- | --- | --- | --- | --- |
| Model with the interaction effect | 0.804 | 169 | -1315.423 | 2 | 0.130 | 0.937 |
| Model without the interaction effect | 0.803 | 171 | -1315.553 |  |  |  |

(c) The inclusion of one outlier (‘mating only’ male) did not change the main finding.

|  | df | χ² value | P value |
| --- | --- | --- | --- |
| Intercept | 1 | 277.930 | <0.001 |
| Initial body length (standardised) | 1 | 0.129 | 0.720 |
| Treatment | 2 | 14.523 | <0.001 |

**5-2 Total distance swum**

(a) No effect of different levels of mating costs (excluding one outlier)

|  | df | Sum sq | F value | P value |
| --- | --- | --- | --- | --- |
| Intercept | 1 | 137969694 | 324.018 | <0.001 |
| Initial body length (standardised) | 1 | 67529 | 0.159 | 0.691 |
| Treatment | 2 | 2144 | 0.003 | 0.998 |
| Residuals | 169 | 71961729 |  |  |

(b) Exclusion of the interaction effect (mating treatment*male body size) did not significantly reduce model fit. Therefore, main effects reported from reduced model.

|  | Res.df | RSS | df | Sum sq | F value | P value |
| --- | --- | --- | --- | --- | --- | --- |
| Model with the interaction effect | 167 | 69629078 |  |  |  |  |
| Model without the interaction effect | 169 | 71961729 | -2 | -2332651 | 2.797 | 0.064 |

(c) The inclusion of one outlier (naïve male) resulted in a significant interaction effect.

|  | df | Sum sq | F value | P value |
| --- | --- | --- | --- | --- |
| Intercept | 1 | 138484655 | 312.317 | <0.001 |
| Initial body length (standardised) | 1 | 248746 | 0.561 | 0.455 |
| Treatment | 2 | 95321 | 0.108 | 0.898 |
| Treatment*initial body length | 2 | 3366523 | 3.796 | 0.024 |
| Residuals | 168 | 74492901 |  |  |

**5-3 Time spent with female**

(a) No effect of different levels of mating costs including outliers

|  | df | Sum sq | F value | P value |
| --- | --- | --- | --- | --- |
| Intercept | 1 | 8468418 | 357.686 | <0.001 |
| Initial body length (standardised) | 1 | 1148 | 0.049 | 0.826 |
| Treatment | 2 | 35312 | 0.746 | 0.476 |
| Residuals | 170 | 4024843 |  |  |

(b) Exclusion of the interaction effect (mating treatment*male body size) did not significantly reduce model fit. Therefore, main effects reported from reduced model.

|  | Res.df | RSS | df | Sum sq | F value | P value |
| --- | --- | --- | --- | --- | --- | --- |
| Model with the interaction effect | 168 | 4007485 |  |  |  |  |
| Model without the interaction effect | 170 | 4024843 | -2 | -17358 | 0.364 | 0.696 |

**Trait 6. Sperm count**

(a) Treatment*male body size effect (excluding two outliers)

|  | df | Sum sq | F value | P value |
| --- | --- | --- | --- | --- |
| Intercept | 1 | 13563.3 | 36302.280 | <0.001 |
| Initial body length (standardised) | 1 | 1.6 | 4.183 | 0.042 |
| Treatment | 2 | 9.8 | 13.171 | <0.001 |
| Treatment*initial body length | 2 | 4.6 | 6.104 | 0.003 |
| Residuals | 163 | 60.9 |  |  |

(b) The inclusion of two outliers (one naïve and one ‘mating only’ males) did not change the main finding of the interaction effect.

|  | df | Sum sq | F value | P value |
| --- | --- | --- | --- | --- |
| Intercept | 1 | 13862.0 | 36845.815 | <0.001 |
| Initial body length (standardised) | 1 | 2.2 | 5.798 | 0.017 |
| Treatment | 2 | 10.2 | 13.494 | <0.001 |
| Treatment*initial body length | 2 | 4.1 | 5.431 | 0.005 |
| Residuals | 165 | 62.1 |  |  |

**Trait 7. Sperm replenishment rate**

(a) Treatment*male body size effect (excluding three outliers)

|  | Df | Sum sq | F value | P value |
| --- | --- | --- | --- | --- |
| Intercept | 1 | 11023.1 | 12850.999 | <0.001 |
| Initial body length (standardised) | 1 | 4.6 | 5.305 | 0.0226 |
| Treatment | 2 | 5.5 | 3.183 | 0.044 |
| Treatment*initial body length | 2 | 5.7 | 3.307 | 0.039 |
| Residuals | 159 | 136.4 |  |  |

(b) The inclusion of three outliers (one from each treatment) did not change the main finding of the interaction effect.

|  | Df | Sum sq | F value | P value |
| --- | --- | --- | --- | --- |
| Intercept | 1 | 11276.5 | 12677.128 | <0.001 |
| Initial body length (standardised) | 1 | 5.1 | 5.728 | 0.018 |
| Treatment | 2 | 5.2 | 2.898 | 0.058 |
| Treatment*initial body length | 2 | 5.7 | 3.210 | 0.043 |
| Residuals | 162 | 144.1 |  |  |

**Trait 8. Sperm velocity (VCL)**

(a) No treatment*male body size effect (excluding one outlier)

|  | Df | Sum sq | F value | P value |
| --- | --- | --- | --- | --- |
| Intercept | 1 | 705427 | 3440.550 | <0.001 |
| Initial body length (standardised) | 1 | 314 | 1.531 | 0.218 |
| Treatment | 2 | 139 | 0.338 | 0.714 |
| Treatment*initial body length | 2 | 173 | 0.422 | 0.657 |
| Residuals | 165 | 33830 |  |  |

(b) No effect of different levels of mating costs (excluding one outlier)

|  | Df | Sum sq | F value | P value |
| --- | --- | --- | --- | --- |
| Intercept | 1 | 706634 | 3470.472 | <0.001 |
| Initial body length (standardised) | 1 | 230 | 1.130 | 0.289 |
| Treatment | 2 | 149 | 0.365 | 0.695 |
| Residuals | 167 | 34003 |  |  |

(c) Exclusion of the interaction effect (mating treatment*male body size) did not significantly reduce model fit. Therefore, main effects reported from reduced model.

|  | Res.df | RSS | df | Sum sq | F value | P value |
| --- | --- | --- | --- | --- | --- | --- |
| Model with the interaction effect | 165 | 33830 |  |  |  |  |
| Model without the interaction effect | 167 | 34003 | -2 | -172.96 | 0.422 | 0.657 |

(d) The inclusion of one outlier (naïve male) did not change the main finding.

|  | df | Sum sq | F value | P value |
| --- | --- | --- | --- | --- |
| Intercept | 1 | 707258 | 3256.812 | <0.001 |
| Initial body length (standardised) | 1 | 536 | 2.466 | 0.118 |
| Treatment | 2 | 55 | 0.126 | 0.881 |
| Residuals | 168 | 36483 |  |  |
